# Adherence to Artemisinin-Based Combination Therapy for the Treatment of Uncomplicated Malaria: A Systematic Review and Meta-Analysis

**DOI:** 10.1155/2015/189232

**Published:** 2015-05-28

**Authors:** Ahmad M. Yakasai, Muhammad Hamza, Mahmood M. Dalhat, Musa Bello, Muktar A. Gadanya, Zuwaira M. Yaqub, Daiyabu A. Ibrahim, Fatimah Hassan-Hanga

**Affiliations:** ^1^Infectious and Tropical Diseases Unit, Public Health and Diagnostic Institute, College of Medical Sciences, Northwest University, PMB 3220, Kano, Nigeria; ^2^Infectious and Tropical Diseases Unit, Department of Medicine, Aminu Kano Teaching Hospital, Bayero University, PMB 3452, Kano, Nigeria; ^3^Department of Community Medicine, Aminu Kano Teaching Hospital, Bayero University, PMB 3452, Kano, Nigeria; ^4^Department of Pharmacy, Aminu Kano Teaching Hospital, PMB 3452, Kano, Nigeria; ^5^Department of Medicine, Aminu Kano Teaching Hospital, Bayero University, PMB 3452, Kano, Nigeria; ^6^Infectious and Tropical Diseases Unit, Department of Paediatrics, Aminu Kano Teaching Hospital, Bayero University, PMB 3452, Kano, Nigeria

## Abstract

Adherence to artemisinin-based combination therapy (ACT) is not clearly defined. This meta-analysis determines the prevalence and predictors of adherence to ACT. Twenty-five studies and six substudies met the inclusion criteria. The prevalence of ACT adherence in the public sector was significantly higher compared to retail sector (76% and 45%, resp., *P* < 0.0001). However, ACT adherence was similar across different ACT dosing regimens and formulations. In metaregression analysis prevalence estimates of adherence significantly decrease with increasing year of study publication (*P* = 0.046). Factors found to be significant predictors of ACT adherence were years of education ≥ 7 {odds ratio (OR) (95% CI) = 1.63 (1.05–2.53)}, higher income {2.0 (1.35–2.98)}, fatty food {4.6 (2.49–8.50)}, exact number of pills dispensed {4.09 (1.60–10.7)}, and belief in traditional medication for malaria {0.09 (0.01–0.78)}. The accuracy of pooled estimates could be limited by publication bias, and differing methods and thresholds of assessing adherence. To improve ACT adherence, educational programs to increase awareness and understanding of ACT dosing regimen are interventions urgently needed. Patients and caregivers should be provided with an adequate explanation at the time of prescribing and/or dispensing ACT.

## 1. Introduction

Malaria is endemic in several countries in Africa, Asia, and South America. It is associated with high morbidity and mortality especially in children under five years of age. The economic loss due to malaria infection is huge in malaria endemic regions. It has been estimated that, in some countries of Sub-Saharan Africa (SSA), up to 10% of monthly income could be spent on procuring a complete dose of ACT for a child [1, 2]. The epidemic of malaria has been fuelled by widespread resistance to cheap antimalarial drugs like chloroquine. Thus, there was an urgent need to shift from monotherapy to combination therapy in order to prevent further development of resistance [3]. In 2001, the World Health Organization (WHO) recommended artemisinin-based combination therapy (ACT) as the first line therapy for uncomplicated malaria [3]. Since then adoption of ACT in malaria endemic countries especially in SSA has been slow due to the lack of understanding of the concept of ACT, its fundamental principles, and its high cost. Other problems relate to the nonavailability of appropriate paediatric formulations and the erratic supply of ACTs in endemic areas. Adoption of ACT as the first line treatment for uncomplicated malaria could have positive impact on individuals, their families, and the community at large by reducing incidence of complications, preventing development of multidrug resistant malaria species and reducing the socioeconomic burden of malaria infection. In addition ACT reduces morbidity and mortality from malaria indirectly by preventing progression of uncomplicated malaria to severe malaria. ACTs not only are good in treatment of malaria but also serve as tools for prevention and control of malaria due to their ability to reduce the infectivity of mosquitoes especially in areas of low or moderate malaria transmission [4].

Several ACT combinations available include artemether-lumefantrine (AL), artesunate-amodiaquine (AS/AQ), artesunate-mefloquine (AS/MQ), artesunate-chlorproguanil-dapsone (AS/CD), artesunate-sulphadoxine-pyrimethamine (AS/SP), dihydroartemisinin-piperaquine (DA/PQ), artesunate-piperazine (AS/PZ), and artesunate-atovaquone-proguanil (A/AP) [5]. Out of these ACT combinations WHO recommended AL, AS/MQ, AS/AQ, and AS/SP [6]. Several countries have now adopted ACTs as the first line agents for uncomplicated malaria [7]. However, a major challenge with ACT is that because of the rapid response and resolution of symptoms patients or caregivers of children tend to terminate treatment prematurely keeping the remaining tablets for future malaria episodes [8, 9]. Studies conducted across the world have reported varying levels of adherence to ACT both in the public and retail sectors. Moreover, two recently published reviews also noted this wide variation in adherence to ACT [10, 11]. This meta-analysis was therefore conducted to derive pooled estimates of prevalence and predictors of adherence to ACTs since adherence has been shown to be a key challenge in the implementation and adoption of ACT [12].

## 2. Materials and Methods

### 2.1. Process of Article Search and Selection Criteria

Peer-reviewed articles published in English language were searched for electronically in Google Scholar, MEDLINE, EMBASE, AJOL, Web of Science, Cochrane database, and other relevant web sites. Infectious diseases journals, malaria journals, and public health journals were also searched. Medical Subject Heading (MeSH) terms applied in different combinations include “Malaria,” “adherence,” “non adherence,” “predictors,” “risk factors,” “artemisinin,” “antimalarial,” and “treatment.” Search was conducted up to April 25th, 2015. All studies that satisfied the following criteria were included.

1. Reported prevalence and/or predictors of adherence to ACT given for the treatment of uncomplicated malaria.
2. Reported method of assessing adherence.

Two reviewers independently extracted information from the articles. Disagreements were resolved by mutual consensus and/or consultation of third reviewer.

### 2.2. Assessment of Study Quality

The Downs and Black checklist was used to assess methodological quality of included studies [13]. This meta-analysis was conducted and reported according to the guidelines of “Preferred Reporting Items for Systematic Reviews and Meta-Analysis” (PRISMA) and “Meta-Analysis of Observational Studies in Epidemiology” (MOOSE) [14, 15].

### 2.3. Data Analysis

For each of the included studies we recorded the prevalence of adherence to ACT and the 95% Confidence Interval (CI), the standard error, the log of the prevalence, the odds ratio of adherence, and the log of odds ratio. DerSimonian and Laird meta-analyses were performed on the prevalence and odds ratio of adherence to ACT [16]. The level of statistical heterogeneity was used to select an appropriate model for the meta-analysis. Specifically, where we encountered significant heterogeneity (*I* ^2^ statistics >50%), a random effects model (REM) is applied whereas if heterogeneity is not significant we apply a fixed effects model (FEM). Publication bias was assessed with Begg's and Egger's tests [17, 18]. Because these tests could be inconsistent and sometimes a funnel plot could be misleading, we considered publication bias to be present if both tests were able to detect it [19, 20]. Sensitivity analysis was done to examine if any of the studies exerts profound influence on meta-analysis estimates [21]. Quality assessment of included studies was also done. Stata version 12 (Stata Corp., College Station, TX, USA) was used for data analysis.

### 2.4. Characteristics of Studies Included in the Meta-Analysis

**Figure 1:**
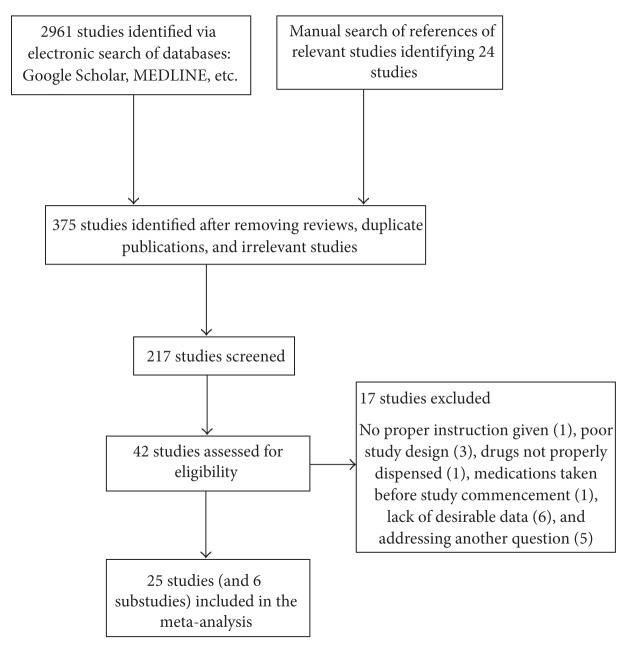
Flow diagram for selecting studies for review and meta-analysis.

As shown in Figure 1, thirty-one studies (twenty-five main studies and six substudies) [12, 22–45] met the inclusion criteria and their characteristics are shown in Table 1. Twenty-two of the studies (and three substudies) were done in the public sector where medications were given for free and dispensed by health professionals with clear instructions [22–35, 37, 39–45]. The other six studies were done in the retail sector where patients or caregivers buy medications from drug stores or supermarkets without proper instructions on how to take them [12, 36, 38, 40]. All the studies included in the systematic review and meta-analysis had satisfactory data quality as shown in Table 2. Study subjects were mainly children, adolescents, and adults. Questions on adherence to ACT were responded to by adults, adolescents, and caregivers of children.

## 3. Results

### 3.1. Public Sector versus Retail Sector

**Figure 2:**
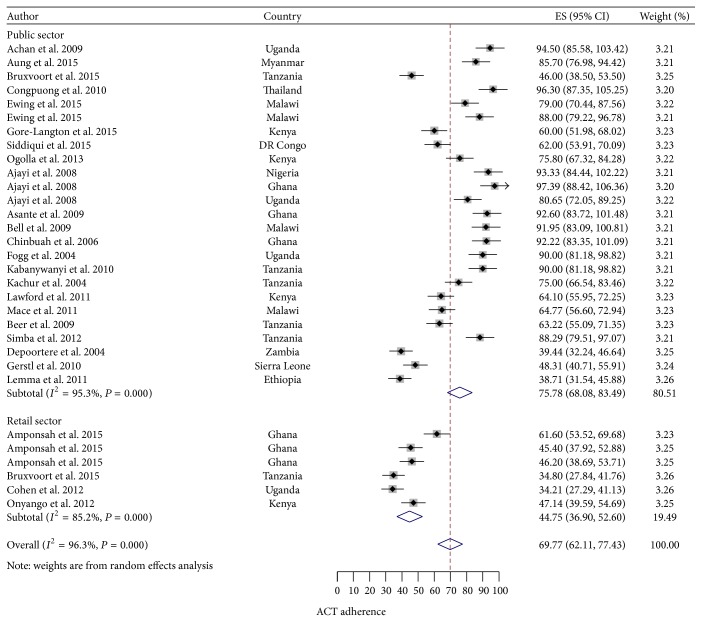
Forest plot of prevalence of adherence in the public and retail sectors.

As indicated in Figure 2, using REM the pooled prevalence (95% CI) of adherence to ACT from the twenty-five studies done in the public sector was 75.78% (68.08%–83.49%). Significant statistical heterogeneity was present (*I* ^2^ = 95.3%, *P* < 0.0001). There was publication bias indicated by significant *P* values in both Begg's and Egger's tests (0.002 and 0.012, resp.). Sensitivity analysis showed that none of the studies exert profound influence on the derived estimates of adherence. From the six studies done in the retail sector the REM pooled prevalence (95% CI) of adherence to ACT was 44.75% (36.90%–77.43%). Significant statistical heterogeneity was present (*I* ^2^ = 96.3%, *P* < 0.001). No publication bias was detected (Begg's test *P* value = 0.851 and Egger's test *P* value = 0.699).

### 3.2. AL versus AS-AQ

When meta-analysis was restricted to the eighteen studies (and two sub-studies) [12, 22, 24, 25, 28–31, 34–40, 42, 43, 45] that administered AL, the REM pooled prevalence of adherence (95% CI) was 70.52% (60.82%–80.22%). No publication was detected (Begg's test *P* value = 0.056 and Egger's test *P* value = 0.018). From the six studies [22, 23, 26, 27, 38, 44] that administered AS-AQ, the REM pooled prevalence of adherence (95%CI) was 68.05% (50.90%–85.19%). No publication bias was detected (Begg's and Egger's tests *P* values were 0.133 and 0.003, resp.).

### 3.3. High versus Low to Moderate Malaria Transmission Intensity Areas

From the 13 studies (and 5 substudies) [22, 23, 26, 28, 30, 31, 34, 35, 38, 39, 41, 42, 44] performed in areas with high malaria transmission intensity, the prevalence (95% CI) of ACT adherence was 77.88% (69.08%–86.67%). There was publication bias (Begg's and Egger's tests *P* values were 0.002 and 0.001, resp.). The pooled prevalence (95% CI) of ACT adherence from the ten studies (and one substudy) [12, 24, 27, 29, 32, 33, 36, 37, 40, 45] performed in areas with low to moderate malaria transmission intensity was 55.58% (44.23%–66.94%) with nonsignificant *P* values in both Begg's and Egger's tests (0.640 and 0.237, resp.).

### 3.4. Twice Daily versus Once Daily ACTs

From the eighteen studies (and two substudies) [12, 22–25, 28–31, 34–38, 40, 42, 43, 45] that used twice daily ACT, the prevalence of adherence was 69.33% (59.57%–79.09%). There was publication bias (Begg's test *P* value = 0.022 and Egger's test *P* value = 0.011). From the ten studies [22, 26, 27, 32, 33, 38, 41, 42, 44] that used once daily ACT, the prevalence of adherence was 66.01% (52.72%–79.29%) with no publication bias (Begg's test *P* value = 0.074 and Egger's test *P* value = 0.001).

### 3.5. Copacked versus Fixed Drug Combination ACTs

The prevalence of adherence to ACT derived from the eleven studies (and one substudy) [22, 23, 26, 27, 32, 33, 38, 40–42, 44] that administered copackaged ACT was 66.53% (54.23%–78.83%). There was no publication bias (Begg's test *P* value = 0.075 and Egger's test *P* value = 0.008). The prevalence of adherence from the nineteen studies (and two substudies) [12, 22, 24, 25, 28–31, 34–40, 42–45] that administered fixed drug combination ACT was 70.11% (60.88%–79.34%). There was no publication bias (Begg's test *P* value = 0.070 and Egger's test *P* value = 0.016).

### 3.6. Metaregression

**Figure 3:**
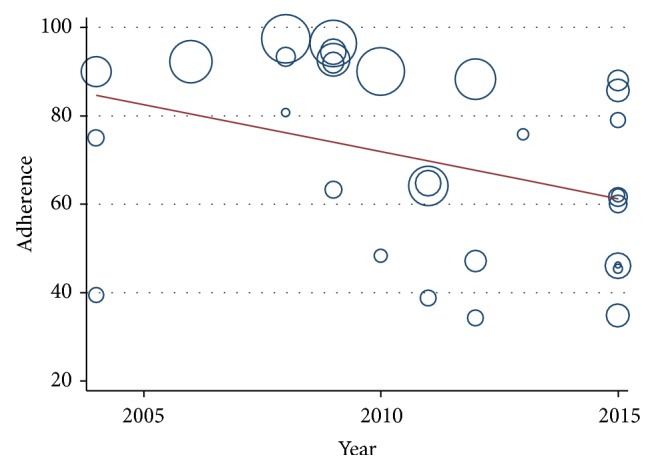
Metaregression of ACT adherence estimate by year of study publication.

In metaregression analysis the prevalence of adherence to ACT significantly decreases with increasing year of publication as shown in Figure 3 (*P* = 0.046, 95% CI = −4.223 to −0.036).

### 3.7. Predictors of ACT Adherence

**Figure 4:**
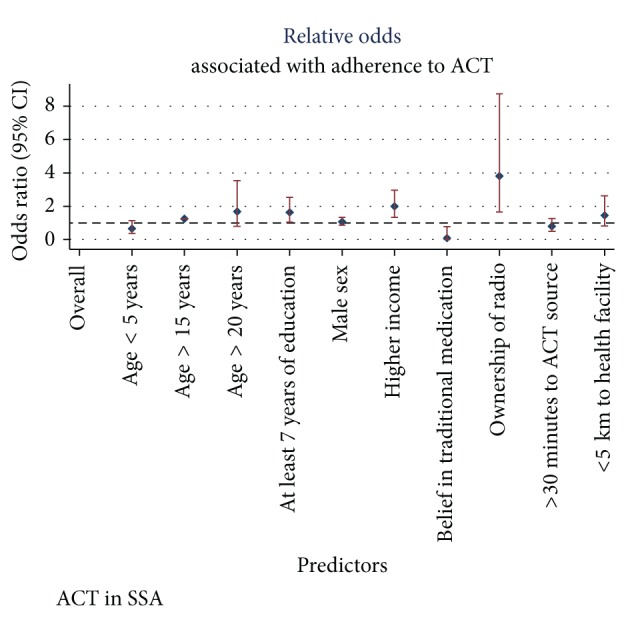
Sociodemographic predictors of ACT adherence.

**Figure 5:**
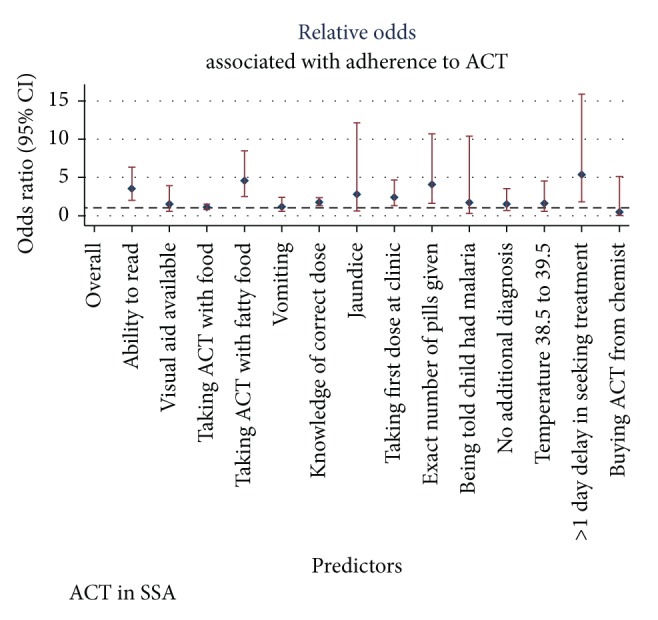
Clinical and dispensing-related predictors of ACT adherence.

These are shown in Figures 4 and 5.

## 4. Discussion

This meta-analysis found significantly higher level of adherence to ACT in the public sector as compared to retail sector (76% and 45%, resp., *P* < 0.0001). This is not unexpected as the two sectors differ in functions, structure, and quality of services offered. In the public sectors, ACTs are usually given for free and health professionals offer clear instructions on how to take medications. In this meta-analysis some of the included studies done in the public sector provided ACTs with pictorial instruction. This may enhance compliance and ingestion of correct dosages at the right time. On the other hand the retail sector observations reflect the real life situation in most communities in malaria endemic countries where patients or caregivers of children commonly purchase ACTs from informal private outlets such as drug stores, pharmacies, and supermarkets. These private outlets may or may not be licensed to sell drugs and about 80% of malaria episodes commonly receive antimalarial therapy in these settings [46]. Diagnoses of malaria in these settings most of the time are presumptive and drugs with questionable potency are dispensed without clear instructions. In drug shops, presumptive treatment of malaria is one of the factors identified to be responsible for the inappropriate ACT treatment of most cases [47]. The reported efficacy of ACTs from clinical trials may be difficult to maintain in communities with poor ACT adherence, inappropriate drug policies, substandard drugs, and poor supervision and monitoring by drug regulatory agencies.

This meta-analysis found poor adherence in children less than five years of age which may be related to the lack of appropriate dose formulations for this vulnerable age group [30]. This is a major threat to global efforts to roll back malaria. The probability of developing drug resistant malaria species is higher in children less than five years of age than in adults because of higher parasite biomass in children [36]. In places of low intensity malaria transmission, poorly or incompletely treated malaria infections in the presence of a high parasite biomass could promote development of* de novo* resistance to antimalarial drugs [48]. Due to the high cost of ACT, parents or caregivers of children may favour traditional medication over ACT. Moreover, coadministration of ACT with traditional medication could lead to treatment interruption, subtherapeutic drug concentration, poor parasitological and clinical response, and nonadherence. Findings from this meta-analysis indicate that lower socioeconomic status (SES) predicts poor adherence to ACT. Children from poorer families may not receive optimum treatment and care during episodes of malaria infection as compared to children from richer families [49]. The role of SES in ACT adherence is multidimensional because some of the factors associated with good adherence to ACT from this meta-analysis may be directly or indirectly related to SES. These factors include ability to read, ownership of radio, and education. Other factors we found to be associated with good adherence to ACT include instructing patients or caregivers to ingest ACT with fatty food, receiving the exact number of pills required for full treatment, and knowledge of correct ACT dose. These dispensing-related factors could positively influence adherence where the pharmacists or healthcare workers routinely practice them while dispensing ACT. Proper communication skills could promote adherence to ACT as we observed in this meta-analysis. Informing caregivers or parents that their child had malaria and availability of visual aid to enhance understanding of dosing instructions were associated with good adherence. Severity of malaria at presentation may determine the level of adherence as we found that patients with jaundice and high body temperature at presentation had good adherence to ACT. Patients, parents, or caregivers of children may perceive these symptoms as serious thereby promoting adherence with treatment instructions.

The cost of ACT is up to 20 times that of previous conventional drugs such as chloroquine and sulphadoxine-pyrimethamine used for the treatment of uncomplicated malaria. The high cost of ACT together with the cost of effecting policy change may have contributed to the delay in adopting ACT as the first line choice for uncomplicated malaria in resource poor settings. Concern over the safety of ACTs had also contributed to delay in adopting it as first line therapy. Neurotoxicity manifesting as hearing loss has been reported with use of coartemether to treat uncomplicated malaria [50]. Moreover, safety of ACT in pregnancy has not been established.

We observed fairly similar levels of adherence to AL and AS/AQ (71% and 80%, resp., *P* = 0.139) in the current meta-analysis. It has been reported that adherence to antimalarial agents is inversely proportional to length of therapy and frequency of taking dosages [51]. The complete treatment of uncomplicated malaria requires a patient to take 6 doses of AL as compared to 3 doses for other ACT-based combinations [5]. Despite the increased dosages of AL over AS/AQ, we did not observe a statistically significant difference in adherence to these antimalarial agents. Further, fairly similar levels of adherence were observed between single and twice daily ACTs in this meta-analysis (66% and 69%, resp., *P* = 0.651). Adherence to copackaged ACTs was also fairly similar to that of fixed drug combination ACTs (67% and 70%, resp., *P* = 0.649). However, it is worthy to note that the accuracy of adherence to copackaged ACTs is limited by publication bias. Further studies are needed to assess the influence of dosing regimen complexity, copackaging, and fixed drug combination on adherence to ACT.

It was observed in this study that poor adherence to ACT was mainly in the last two doses at the end of treatment [25, 28, 35, 45]. This may be related to the rapid resolution of symptoms after the first few doses which caregivers or patients wrongly interpret as cure. For economic reasons some patients may be tempted to keep remaining tablets for anticipated episodes of malaria.

Substandard antimalarial drugs pose a major threat to global efforts to combat malaria. A survey of drugs conducted in 21 countries in SSA found substantial proportion of drugs that failed chemical and packaging analysis while others were falsified [52]. Poor quality ACTs in addition to poor adherence could amplify the rate at which multidrug resistant malaria strains develop [53]. Resistance to artemisinin has already been reported [54, 55] and efforts to curtail this resistance should consider level of poor adherence to ACT in the retail sector, public sector, and areas with low to moderate malaria transmission intensity. It was observed in this meta-analysis that adherence to ACT in areas with high malaria transmission intensity was significantly higher than adherence in areas with low to moderate transmission intensity (78% and 56%, resp., *P* = 0.001). This poor adherence to ACT in areas with low to moderate malaria transmission intensity is a major concern because drug resistant* Plasmodium falciparum* infection may spread more rapidly in this setting [56, 57].

Several caveats should be considered while interpreting the findings from this meta-analysis. Self-report used to assess adherence when blister pack is not available for verification is not a good measure of adherence due to recall bias. Only one study [28] used electronic pill-box monitoring which is a more accurate way of assessing adherence [58]. Different thresholds of adherence were used in the studies and include percentage of correctly ingested doses or number of tablets left on day three. Determination of blood concentration of drugs to confirm adherence was done only in 4 studies [28, 31, 35, 41] and self-report of adherence was found to have a failure rate of 2.4% in one of the studies [41]. Although assaying blood levels of ACTs could increase accuracy of defining adherence, its role in this meta-analysis may be limited by pharmacokinetic differences between children and adults. Variable blood concentrations of ACTs could arise from age- or weight-based dosing and ingestion of lumefantrine with fatty meal (instruction not given in some of the studies). Despite these caveats, restricted analysis reduces heterogeneity and confounding thereby increasing reliability of estimates. The strength of this meta-analysis lies in its large sample size (8654 subjects), exclusion of confounders, and unannounced home visit in most of the studies included (see Table 2). Two recently conducted systematic reviews found wide variability of ACT adherence across different populations [10, 11]. In these reviews there are no pooled estimates of prevalence of adherence and this could undermine their epidemiologic significance. The current study also encountered variability of reported ACT adherence arising from environmental factors. However, effort was made to substantially reduce heterogeneity between studies by meta-analytically pooling estimates from fairly similar populations via restricted analysis.

Currently ACT is the best and recommended first line therapy for uncomplicated malaria in several countries (see Table 3) [7]. To maintain its long term efficacy there is a need to train healthcare providers on appropriate dosing and dispensing of ACT. The poor adherence observed in the retail sector and areas with low to moderate malaria transmission and suboptimal adherence in public sector calls for more efforts to improve adherence to ACT in malaria endemic countries of the world. Barriers to adherence should be addressed because nonadherence could be a platform for the development of antimalarial resistance. At community level people should be educated on the adverse effects and implications of poor adherence to ACT. Training of healthcare providers and adequate supply of subsidized potent ACTs with clear instructions and packaging leaflets in local languages could improve adherence. Furthermore, pictorial instructions could help bridge the communication gap that may exist between health professionals and patients or caregivers.

## 5. Conclusion

Findings from this systematic review and meta-analysis indicated that adherence to ACT in the retail sector and areas with low/moderate malaria transmission are poor. Healthcare providers, policy makers, and other stakeholders involved in malaria control programs should take cognisance of these findings when designing targeted interventions to improve ACT adherence. Educational programs to increase awareness and understanding of ACT dosing regimen are interventions urgently needed to improve adherence to ACT. Patients or caregivers should be provided with an adequate explanation at the time of prescribing and/or dispensing ACT. Prescribers, dispensers, and vendors should, therefore, give a clear and comprehensible explanation on how to use ACT. User-friendly packaging (e.g., blister packs) should be used to encourage completion of the treatment course and correct dosing.

## Figures and Tables

**Table 1:** Characteristics of studies included in the systematic review and meta-analysis.

Author/year	Country	Sample size	Endemicity	ACT	Adherence measure	Comments/sources of bias
Ajayi et al. 2008 [22]	Nigeria	288	High	AL	Blister pack/caregivers recall	>97% of CMDs prescribing correct ACT doses

Ajayi et al. 2008 [22]	Ghana	382	High	AS/AQ	Blister pack/caregivers recall	>97% of CMDs prescribing correct ACT doses

Ajayi et al. 2008 [22]	Uganda	619	High	AL	Blister pack/caregivers recall	>97% of CMDs prescribing correct ACT doses

Asante et al. 2009 [23]	Ghana	401	High	AS/AQ	Blister pack	Home visit to supervise treatment which could influence adherence

Bell et al. 2009 [28]	Malawi	209	High	AL	Questionnaire and electronic device	Blood LU assayed on day 7

Chinbuah et al. 2006 [34]	Ghana	NA	High	AL	Blister pack	Majority of children receiving correct dose of AL

Fogg et al. 2004 [31]	Uganda	210	High	AL	Blister pack	Educated semiurban population, blood lumefantrine level assayed

Kabanywanyi et al. 2010 [25]	Tanzania	522	NR	AL	Blister pack	Pictorial instruction available, questionnaire not validated

Kachur et al. 2004 [32]	Tanzania	253	Moderate	AS/SP	Blister pack/self-report	Socioeconomic status assessed

Lawford et al. 2011 [24]	Kenya	918	Low/moderate	AL	Blister pack/self-report	Validity of translated questionnaire not mentioned

Mace et al. 2011 [30]	Malawi	868	High	AL	Pill count/dose recall	Pictorial instructions provided

Beer et al. 2009 [27]	Tanzania	195	Moderate	AS/AQ	Pill count/self-report	>50% of nonadherence was due to misunderstanding of instructions

Simba et al. 2012 [35]	Tanzania	444	High	AL	Self-report	Plasma LU concentration assayed, pictorial instruction provided, and questionnaire validated

Depoortere et al. 2004 [33]	Zambia	142	Moderate	AS/SP	Blister pack	No adequate dosage instructions

Gerstl et al. 2010 [26]	Sierra Leone	118	High	AS/AQ	Blister pack	37% given wrong dosage instructions

Lemma et al. 2011 [29]	Ethiopia	155	Low	AL	Blister pack/self-report	Study done at peak of malaria transmission

Onyango et al. 2012 [12]	Kenya	297	Low	AL	Pill count/self-report	Covered long distance to get ACT, questionnaire validated

Cohen et al. 2012 [36]	Uganda	152	Low	AL	Blister pack/self-report	Drug shops licensed, ACT heavily subsidized, and shop attendants trained and supervised

Achan et al. 2009 [37]	Uganda	175	Low	AL	Blister pack	Adequate instruction given

Amponsah et al. 2015 [38]	Ghana	219	High	AL	Pill count	Questionnaire validated

Amponsah et al. 2015 [38]	Ghana	55	High	AS/AQ	Pill count	Questionnaire validated

Amponsah et al. 2015 [38]	Ghana	26	High	AS/PP	Pill count	Questionnaire validated

Aung et al. 2015 [39]	Myanmar	161	High	AL	Pill count/self-report	Good dosage instructions given

Bruxvoort et al. 2015 [40]	Tanzania	572	Low	AL	Pill count/self-report	Taking first dose at health facility associated with good adherence

Bruxvoort et al. 2015 [40]	Tanzania	450	Low	AL	Pill count/self-report	Several drug shops not yet accredited and not receiving training

Congpuong et al. 2010 [41]	Thailand	240	High	AS/MQ	Day 3 whole blood MQ concentration and self-report	Self-report failing to identify 2.4% of subjects with poor adherence

Ewing et al. 2015 [42]	Malawi	101	High	AL	Pill count, blister pack, and self-report	Underdosing due to loss of medication, children followed up for 3 years to monitor long term effects of ACT

Ewing et al. 2015 [42]	Malawi	117	High	DA/PQ	Pill count, blister pack, and self-report	Underdosing due to loss of medication, children followed up for 3 years to monitor long term effects of ACT

Gore-Langton et al. 2015 [43]	Kenya	195	Low	AL	Self-report and blister pack	Study area affected by conflict leading to poor health delivery services

Siddiqui et al. 2015 [44]	DR Congo	108	High	AS/AQ	Self-report and blister pack	Poor understanding of the need to complete treatment schedule

Ogolla et al. 2013 [45]	Kenya	62	Low	AL	Pill count, blister pack, and self-report	Malaria was microscopically confirmed. The sixth ACT dose was the most commonly forgotten tablet

ACT: artemisinin-based combination therapy, AL: artemether-lumefantrine, AQ: amodiaquine, AS: artesunate, CMDs: community medicine distributors, DA: dihydroartemisinin, PQ: piperaquine, PP: piperazine, and SP: sulphadoxine-pyrimethamine.

**Table 2:** Quality assessment of included studies.

Author	Adequate sample size	Reported baseline characteristics	Randomization	Unannounced home visit	Reported adherence measure	Addressed lost to follow-up	Excluded confounders
Ajayi et al. 2008 [22]	Y	Y	N	NC	Y	NC	Y

Asante et al. 2009 [23]	Y	Y	Y	NC	Y	Y	Y

Bell et al. 2009 [28]	Y	Y	Y	N	Y	N	Y

Chinbuah et al. 2006 [34]	NA	Y	N	N	Y	NR	N

Fogg et al. 2004 [31]	Y	Y	N	Y	Y	NR	Y

Kabanywanyi et al. 2010 [25]	Y	Y	Y	N	Y	NR	N

Kachur et al. 2004 [32]	Y	Y	Y	Y	Y	NR	Y

Lawford et al. 2011 [24]	Y	Y	N	Y	Y	NR	Y

Mace et al. 2011 [30]	Y	Y	N	Y	Y	Y	Y

Beer et al. 2009 [27]	Y	Y	N	Y	Y	Y	Y

Simba et al. 2012 [35]	Y	Y	N	Y	Y	Y	Y

Depoortere et al. 2004 [33]	Y	Y	N	Y	Y	Y	Y

Gerstl et al. 2010 [26]	Y	Y	N	Y	Y	Y	Y

Lemma et al. 2011 [29]	Y	Y	N	Y	Y	Y	Y

Onyango et al. 2012 [12]	Y	Y	N	NC	Y	NR	Y

Cohen et al. 2012 [36]	Y	Y	Y	Y	Y	Y	Y

Achan et al. 2009 [37]	Y	Y	N	Y	Y	NC	Y

Amponsah et al. 2015 [38]	Y	Y	N	N	Y	Y	Y

Aung et al. 2015 [39]	Y	Y	N	Y	Y	Y	Y

Bruxvoort et al. 2015 [40]	Y	Y	N	NC	Y	Y	Y

Congpuong et al. 2010 [41]	Y	Y	N	N	Y	NR	Y

Ewing et al. 2015 [42]	Y	Y	Y	N	Y	Y	Y

Gore-Langton et al. 2015 [43]	Y	Y	N	N	Y	Y	Y

Siddiqui et al. 2015 [44]	Y	Y	N	N	Y	Y	Y

Ogolla et al. 2013 [45]		Y	N	NC	Y	Y	Y

Y: yes, N: no, NC: not clear, and NR: not reported.

**Table 3:** ACT recommended in different countries [7].

Continent	ACT recommended	Countries
Africa	AL	Angola, Benin, Burkina Faso, CAR, Chad, Comoros, Ethiopia, South Africa, Tanzania, Togo, Zambia, Zimbabwe, Côte d'Ivoire, Djibouti, Gabon, Malawi, Mozambique, Sudan (N), Sao Tome and Principe, and Tanzania
	AS/AQ	Burundi, Cameroon, Côte d'Ivoire, Democratic Republic of the Congo, Equatorial Guinea Gabo, Ghana, Liberia, Madagascar, Malawi, Mauritania, Senegal, Sao Tome and Principe, Sierra Leone, Sudan (S), and Tanzania
	AS/SP	Mozambique, Djibouti, Somalia, South Africa, and Sudan (N)

Asia	AS/MQ	Cambodia, Malaysia, Myanmar, and Thailand
	AL	Bangladesh, Bhutan, Laos, and Saudi Arabia
	AS/AQ	Indonesia
	AS/SP	Afghanistan, India, Iran, Tajikistan, Yemen, and Papua New Guinea
	DP	Vietnam
	AL	Iran, Philippines, and Solomon Islands

South America	AS/SP	Ecuador, Peru
	AS/MQ	Bolivia, Peru, and Venezuela
	AL	Brazil, Guyana, and Suriname

AL: artemether-lumefantrine, AQ: amodiaquine, AS: artesunate, DA/PQ: dihydroartemisinin/piperaquine, MQ: mefloquine, and SP: sulphadoxine-pyrimethamine.
